# The Mental Well-Being of Frontline Physicians Working in Civil Wars Under Coronavirus Disease 2019 Pandemic Conditions

**DOI:** 10.3389/fpsyt.2020.598720

**Published:** 2021-01-14

**Authors:** Muhammed Elhadi, Ahmed Msherghi, Moutaz Elgzairi, Ayiman Alhashimi, Ahmad Bouhuwaish, Marwa Biala, Seraj Abuelmeda, Samer Khel, Ala Khaled, Ahmed Alsoufi, Ahmed Elhadi, Ahmed BenGhatnsh

**Affiliations:** ^1^Faculty of Medicine, University of Tripoli, Tripoli, Libya; ^2^Faculty of Medicine, University of Benghazi, Benghazi, Libya; ^3^Faculty of Medicine, Al-Jabal Al Gharbi University, Gherian, Libya; ^4^Faculty of Medicine, Tobruk University, Tobruk, Libya

**Keywords:** COVID-19, depression, anxiety, burnout, pandemic, pandemic (COVID-19)

## Abstract

**Background and Objective:** Emergency medical physicians are regarded as essential frontline staff in combating the coronavirus disease 2019 (COVID-19) pandemic. These health-care workers are faced with significant stressors in addition to the usual stress felt in their regular work. Therefore, this study aimed to examine the prevalence of anxiety, depression, and burnout among emergency physicians on the frontline of the COVID-19 pandemic.

**Method:** Using a cross-sectional study methodology, we surveyed physicians active on April 2020 to study depression and anxiety [using Hospital Anxiety and Depression Scale (HADS)] and burnout [using the Abbreviated Maslach Burnout Inventory (aMBI) scale].

**Results:** A total of 154 emergency physicians completed the survey. We found that about 65.6% of patients were experiencing anxiety (based on a HADS score ≥ 11), and 73.4% were displaying depressive symptoms. For burnout, three subscales indicated that 67.5% endured emotional exhaustion, and 48.1% experienced depersonalization (defined as a score of ≥10 on aMBI). A total of 21.4% of respondents perceived a sense of personal underachievement, defined as a score of <10 via aMBI.

**Conclusion:** Physicians' psychological status is crucial and plays a major role in their well-being, affecting their work satisfaction. Therefore, implementing strategies aimed at decreasing the impact of stressful events is crucial to alleviate the distress experienced by physicians on the frontline of the COVID-19 pandemic.

## Introduction

The severe acute respiratory syndrome coronavirus 2 (SARS-CoV-2) was identified in late 2019 in Wuhan, China, as the cause of numerous severe viral pneumonia cases (1). On February 11, 2020, the World Health Organization (WHO) designated the name “COVID-19” (coronavirus disease 2019) for the viral pneumonia caused by SARS-CoV-2 (2). In the wake of the announcement, the virus reached pandemic levels, resulting in more than 54 million cases worldwide and more than 1,300,000 deaths by November 15, 2020 (3).

Emergency medical physicians are regarded as frontline staff in combating the COVID-19 pandemic. They are the first contact point for any patients who display signs and symptoms of COVID-19 infection. This is in addition to the usual stress felt by emergency physicians during their work (4).

Previous studies have reported that burnout (a state of emotional exhaustion resulting in the loss of enthusiasm for one's work) and depersonalization (a feeling of cynicism accompanied by a low sense of personal accomplishment) are signs of mental health deterioration. This deterioration has negative consequences for a physician's ability to judge, diagnose, and deliver satisfactory health-care services for their patients (5–7). Furthermore, it has been reported that burnout and high emotional pressure can increase the rates of both medical error and substance abuse; the literature has even indicated an increase in risk of suicide (7–11).

Several studies have investigated the prevalence of psychological distress experienced by health-care workers during the COVID-19 pandemic. However, none have focused on emergency physicians, although they are the first line (12–18), and no studies have been conducted in African regions.

Therefore, understanding the causes and effects of stress and its consequences is critical for the well-being of emergency doctors, as stress and emotional pressure can have negative and deleterious consequences on physicians' well-being and their ability to deliver appropriate health-care services to their patients (19, 20).

Libya, like a number of other countries, is suffering from civil war and financial crises, increasing the risk of mental dysfunction and mental disorders such as depression, anxiety, and burnout (21–23).

Furthermore, the scientific literature lacks data concerning the rate of burnout among emergency physicians, especially those deployed during the COVID-19 pandemic. Including our present knowledge of the state of the literature, there is no recent study concerning emergency physicians' mental status as impacted by the COVID-19 pandemic and foregrounded by civil war. Therefore, in this study, we aimed to examine the prevalence of anxiety, depression, and burnout among emergency physicians dealing with the COVID-19 pandemic on the frontlines.

## Materials and Methods

### Study Design and Setting

This study employed a cross-sectional study methodology.

### Selection of Participants

Between April 18 and 28, 2020, we recruited health-care workers from Libyan hospitals. We only included emergency physicians working during the COVID-19 pandemic. Emergency physicians in Libya are responsible frontline health-care workers for many diseases and conditions such as acute care, urgent toxicological and psychiatric cases, and trauma triage; severe respiratory and cardiovascular disease patients who usually present to the emergency department initially; and suspected COVID-19 patients who have typical COVID-19 symptoms. Therefore, they are at increased risk of contracting a COVID-19 infection and have a higher risk of complex disease. Along with their life risk, as they treat injured and severely disabled war victims and militias, they are usually at higher risk of abuse by militias or their relatives. Our exclusionary criteria comprised those health-care workers who were either retired or on leave during this period, so that they were not involved in the care of patients during the COVID-19 pandemic. We also excluded those with a preexisting mental illness or who failed to complete the psychological data.

### Measurements

Data were collected via a specially designed questionnaire distributed by paper, mobile messaging, and email. The questionnaire consisted of the following categories: demographic and socioeconomic data, mental health assessment, risk of civil war assessment of violence, and assessment of depression and anxiety. Several relevant questions were also chosen to address the prevalence of violent acts enacted against health-care workers and whether such acts were associated with an increase in the risk of depression and anxiety.

### Outcomes and Definition of Outcomes

The Hospital Anxiety and Depression Scale (HADS) was used to measure the prevalence of anxiety and depression among health-care workers (24). The HADS was validated in a previous study as having a mean of 0.83 (Cronbach's alpha) across several languages and different settings, with a sensitivity and specificity of 0.8 (25). The HADS's corresponding questionnaire consists of 14 self-reported items: seven questions concerning anxiety and seven concerning depression. According to Zigmond and Snaith, a score of <7 is considered normal, a score of 8–10 indicates borderline or doubtful cases, and a score of ≥11 indicates definite cases (26).

The second section employed the Abbreviated Maslach Burnout Inventory (aMBI), a nine-item scale used to assess the level of burnout among physicians (27–30). The inventory has three subscales: emotional exhaustion (EE—emotional depletion due to the demands of the job and continuous work-related stress), depersonalization (DP—an impersonal response toward service recipients), and personal accomplishment (PA—the degree of personal competence, achievement, and satisfaction with one's work). The three elements of the subscale were assessed, along with the three items. A seven-point Likert scale ranging from never (0) to every day (6) was used for each item. Therefore, the cumulative score of each subscale ranges from a minimum of 0 to a maximum of 18, which is calculated for each health-care worker surveyed.

For emotional exhaustion and depersonalization, higher scores indicate greater levels of burnout; for personal achievement, the scale is inversed. The internal reliability of each subscale was calculated using Cronbach's alpha coefficient (31, 32) For EE and DP, a subscale score of 0–9 is categorized as “no to low burnout,” whereas a subscale score of 10–18 is regarded as “moderate to severe burnout”; for PA, the scale is inversed.

**Figure 1:**
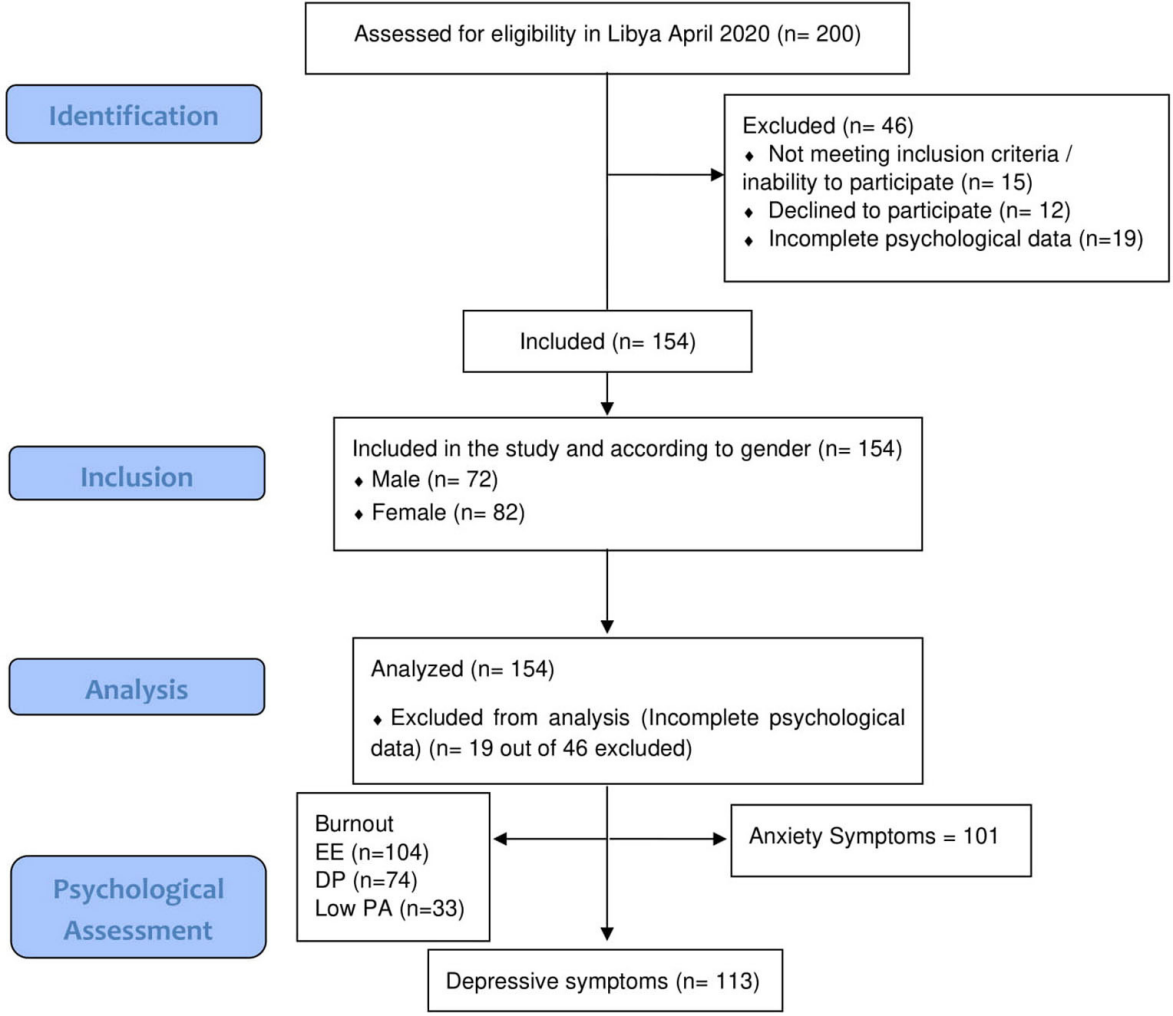
STROBE flowchart. STROBE, Strengthening the Reporting of Observational Studies in Epidemiology.

The reporting of the study follows the Strengthening the Reporting of Observational Studies in Epidemiology (STROBE) statement (33). See flowchart in Figure 1.

Statistical analysis was performed using IBM's SPSS Statistics package for Windows (Version 25.0). Frequency, means, and standard deviations were used to describe the data. Chi-square tests were used to compare the categorical variables, while independent *t*-tests were used to compare continuous variables. Ethical approval for this study was obtained from the Bioethics Committee at the Biotechnology Research Center in Libya.

## Results

### Characteristics of Study Subjects

A total of 154 out of 200 emergency physicians completed the survey, resulting in an estimated 77% response rate; 53.2% of respondents were female. The mean age of emergency physicians surveyed was 31.66 ± 5.97, with ~92.2% of respondents falling below the age of 40 years. About half (51.9%) of the respondents were emergency doctor residents working only within government departments. The majority (69.5%) had <5 years of clinical experience. In terms of the length of their work week, we found that participants worked an approximate mean of 52.95 ± 9.71 h/week, with a mean of 3.68 ± 0.685 night shifts per month for emergency physicians. Table 1 provides an overview of the basic characteristics of emergency medicine physicians who participated in the study.

**Table 1:** Basic characteristics of emergency medicine physicians (*n* = 154).

**Variables**	**Counts *n* = 154**	**Proportions (%)**
**Age range**		
- <40 - ≥40	142 12	92.2 7.8
**Gender**		
- Male - Female	72 82	46.8 53.2
**Marital status**		
- Married	59	38.3
- Not married (including single, divorced, and widowed)	95	61.7
**Residency**		
- Live with family - Live alone	100 54	64.9 35.1
**Employment sector**		
- Governmental only - Private only - Both	80 21 53	51.9 13.6 34.4
**Years of experience**		
- <3 years - 3–5 years - 5–15 - >15 years	73 33 38 9	48.1 21.4 24.7 5.8
**Smoking**		
- Yes - No	27 127	17.5 82.5
**Illicit drug use**		
- Yes - No	8 146	5.2 94.8
**Internally displaced**		
- Yes - No	46 108	29.9 70.1
**Transport issues**		
- Yes - No	57 97	37 63
**Verbal abuse**		
- Yes - No	71 83	46.1 53.9
**Physical abuse**		
- Yes - No	19 135	12.3 87.7

### Main Results

With respect to the prevalence of anxiety and depression, our data based on the HADS indicated that ~65.6% of subjects (*n* = 101) were experiencing anxiety (those who received a score ≥ 11), and about 73.4% of subjects (*n* = 113) were experiencing depressive symptoms (those who received a score ≥ 11). Our data demonstrated that 67.5% (*n* = 104) of subjects suffered from emotional exhaustion, while 48.1% (*n* = 74) experienced depersonalization (both derived from scores of ≥10 out of 18 on the aMBI). However, for low personal accomplishment (PA), only 21.4% (*n* = 33) scored <10 (indicating burnout for this category).

About 46.1% (*n* = 71) of respondents had encountered at least one episode of verbal abuse, while 12.3% (*n* = 19) had experienced physical abuse.

Tables 2–4 depict the association between the basic characteristics and occurrences of anxiety, depression, and burnout among emergency physicians. With the use of a univariate analysis, a comparison between the groups of physicians experiencing anxiety (HADS anxiety ≥ 11) and depression (HADS depression ≥ 11) demonstrated the following elements to be statistically significant: for anxiety, only age range, working hours per week, and transport issues were associated with a higher prevalence of anxiety (*p* < 0.05); however, for depression, none of the demographic and clinical characteristics were significantly associated with a higher prevalence of depression among groups (Table 3).

**Table 2:** Summary of anxiety, depression, and burnout among emergency physicians (*n* = 154).

**Category**	**Grade**	**Mean ± SD**	**Count**	**Percent**
**Depression**	**Total** Normal (0–7) Borderline abnormal (8–10) Abnormal depressive case (11–21)	12.39 ± 2.95	154 7 34 113	100.0 4.5 22.1 73.4
**Anxiety**	**Total** Normal (0–7) Borderline abnormal (8–10) Abnormal anxiety case (11–21)	11.91 ± 3.81	154 21 32 101	100.0 13.6 20.8 65.6
**Burnout**	Emotional exhaustion (EE) ≥ 10 Depersonalization (DP) ≥ 10 Low personal accomplishment (PA) < 10	11.2 ± 5.15 8.55 ± 5.08 12.61 ± 3.89	104 74 33	67.5 48.1 21.4

**Table 3:** Association of depression and anxiety with other characteristics of participants.

**Variables**	**Total**	**Anxiety (+)**	**Anxiety (–)**	***p*-value**	**Depression (+)**	**Depression (–)**	***p*-value**
Total prevalence, n (%)	154	101 (65.6)	53 (34.4)		113 (73.4)	41 (26.6)	
Age range				0.014^*^			0.22
- <40	142	97 (96)	45 (84.9)		106 (93.8)	36 (87.7)	
- ≥40	12	4 (4)	8 (15.1)		7 (6.2)	5 (12.2)	
Gender				0.678			0.761
- Male	82	46 (45.5)	26 (49.1)		52 (46)	20 (48.8)	
- Female	72	55 (54.5)	27 (50.9)		61 (54)	21 (51.2)	
Working hours per week, mean ± SD	52.95 ± 9.71	53.36 ± 10.89	52.19 ± 6.98	0.029^*^	52.73 ± 10.18	53.56 ± 8.37	0.164
Night shifts per month, mean ± SD	3.68 ± 0.68	3.65 ± 0.69	3.72 ± 0.66	0.466	3.65 ± 0.72	3.73 ± 0.549	0.067
Marital status				0.915			0.39
- Married	95	39 (38.6)	20 (37.7)		41 (36.3)	18 (43.9)	
- Not-married (including single, divorced, and widowed)	59	62 (61.4)	33 (62.3)		72 (63.7)	23 (56.1)	
Residency				0.835			0.812
- Live with family	100	36 (35.6)	35 (66)		74 (65.5)	26 (63.4)	
- Live alone	54	65 (64.4)	18 (34)		39 (34.5)	15 (36.6)	
Employment sector				0.929			0.976
- Governmental only	80	53 (52.5)	27 (50.9)		59 (52.2)	21 (51.2)	
- Private only	21	13 (12.9)	8 (15.1)		15 (13.3)	6 (14.6)	
- Both	53	35 (34.7)	18 (34)		39 (34.5)	14 (34.1)	
Years of experience				0.219			0.806
- <3 years	74	50 (49.5)	24 (45.3)		56 (49.6)	18 (43.9)	
−3–5 years	33	22 (21.8)	11 (20.8)		25 (22.1)	8 (19.5)	
−5–5	38	26 (25.7)	12 (22.6)		26 (23)	12 (29.3)	
- >15 years	9	3 (3)	6 (11.3)		6 (5.3)	3 (7.3)	
Smoking				0.227			0.178
- Yes	27	86 (85.1)	12 (22.6)		17 (15)	10 (24.4)	
- No	127	15 (14.9)	41 (77.4)		96 (85)	31 (75.6)	
Illicit drug use				0.85			0.915
- Yes	8	5 (5)	3 (5.7)		6 (5.3)	2 (4.9)	
- No	146	96 (95)	50 (94.3)		107 (94.7)	39 (95.1)	
Internally displaced				0.758			0.764
- Yes	46	31 (30.7)	15 (28.3)		33 (29.2)	13 (31.7)	
- No	108	70 (69.3)	38 (71.7)		80 (70.8)	28 (68.3)	
Transport issues				0.02^*^			0.657
- Yes	57	44 (43.6)	13 (24.5)		43 (38.1)	14 (34.1)	
- No	97	57 (56.4)	40 (75.5)		70 (61.9)	27 (65.9)	
Verbal abuse				0.242			0.487
- Yes	71	50 (49.5)	21 (39.6)		54 (47.8)	17 (41.5)	
- No	83	51 (50.5)	32 (60.4)		59 (52.2)	24 (58.5)	
Physical abuse				0.427			0.557
- Yes	19	14 (13.9)	5 (9.4)		15 (13.3)	4 (9.8)	
- No	135	87 (86.1)	48 (90.6)		98 (86.7)	37 (90.2)	

*The definitive diagnosis of depression or depression was defined as a score of 11–21 on the HADS inventory*.

* *Significant at p < 0.05*.

**Table 4:** Association of burnout with other characteristics of participants.

**Variables**	**Total**	**EE (+)**	**EE (–)**	***p*-value**	**D (+)**	**D (–)**	***p*-value**	**PA (low)**	**PA (high)**	***p-*value**
Total prevalence, n (%)	154	104 (67.5)	50 (32.5)		74 (48.1)	80 (51.9)		33 (21.4)	121 (78.6)	
Age range				0.177			0.645			0.075
- <40	142	98 (94.2)	44 (88)		69 (93.2)	73 (91.3)		28 (84.8)	114 (94.2)	
- ≥40	12	6 (5.8)	6 (12)		5 (6.8)	7 (8.8)		5 (15.2)	7 (5.8)	
Gender				0.412			0.081			0.866
- Male	82	51 (49)	21 (42)		40 (54.1)	32 (40)		15 (45.5)	57 (47.1)	
- Female	72	53 (51)	29 (58)		34 (45.9)	48 (60)		18 (54.4)	64 (52.9)	
Working hours per week, mean ± SD	52.95 ± 9.71	53.53 ± 11.07	51.76 ± 5.92	0.001^*^	53.89 ± 10.67	52.09 ± 8.27	0.087	53.36 ± 6.65	52.84 ± 10.42	0.102
Night shifts per month, mean ± SD	3.68 ± 0.68	3.70 ± 0.736	3.62 ± 0.56	0.612	3.68 ± 0.57	3.68 ± 0.77	0.125	3.73 ± 0.517	3.66 ± 0.72	0.089
Marital status				0.174			0.654			0.885
- Married	95	36 (34.6)	23 (46)		27 (36.5)	32 (40)		13 (39.4)	46 (38)	
- Not married (including single, divorced, and widowed)	59	68 (65.4)	27 (54)		47 (63.5)	48 (60)		20 (60.6)	75 (62)	
Residency				0.049^*^			0.986			0.318
- Live with family	100	73 (70.2)	27 (54)		48 (64.9)	52 (65)		19 (57.6)	81 (66.9)	
- Live alone	54	31 (29.8)	23 (46)		26 (35.1)	28 (35)		14 (42.4)	40 (33.1)	
Employment sector				0.719			0.15			0.192
- Governmental only	80	52 (50)	28 (56)		33 (44.6)	47 (58.8)		21 (63.6)	59 (48.8)	
- Private only	21	14 (13.5)	7 (14)		10 (13.5)	11 (13.8)		5 (15.2)	16 (13.2)	
- Both	53	38 (36.5)	15 (30)		31 (41.9)	22 (27.5)		7 (21.2)	46 (38)	
Years of experience				0.039^*^			0.258			0.001^*^
- <3 years	74	54 (51.9)	20 (40)		40 (54.1)	34 (42.5)		9 (27.3)	65 (53.7)	
−3–5 years	33	17 (16.3)	16 (32)		11 (14.9)	22 (27.5)		14 (42.4)	19 (15.7)	
−5–15	38	29 (27.9)	9 (18)		19 (25.7)	19 (23.8)		6 (18.2)	32 (26.4)	
- >15 years	9	4 (3.8)	5 (10)		4 (5.4)	5 (6.3)		4 (12.1)	5 (4.1)	
Smoking				0.424			0.39			0.912
- Yes	27	20 (19.2)	7 (14)		15 (20.3)	12 (15)		6 (18.2)	21 (17.4)	
- No	127	84 (80.8)	43 (86)		59 (79.7)	68 (85)		27 (81.8)	100 (82.6)	
Illicit drugs use				0.643			0.91			0.8
- Yes	8	6 (5.8)	2 (4)		4 (5.4)	4 (5)		2 (6.1)	6 (5)	
- No	146	98 (94.2)	48 (96)		70 (94.6)	76 (95)		31 (93.9)	115 (95)	
Internally displaced				0.981			0.307			0.951
- Yes	46	31 (29.8)	15 (30)		25 (33.8)	21 (26.3)		10 (30.3)	36 (29.8)	
- No	108	73 (70.2)	35 (70)		49 (66.2)	59 (73.8)		23 (69.7)	85 (70.2)	
Transport issues				0.002			0.124			0.368
- Yes	57	47 (45.2)	10 (20)		32 (43.2)	25 (31.3)		10 (30.3)	47 (38.8)	
- No	97	57 (54.8)	40 (80)		42 (56.8)	55 (68.8)		23 (69.7)	74 (61.2)	
Verbal abuse				0.162			0.057			0.632
- Yes	71	52 (50)	19 (38)		40 (54.1)	31 (38.8)		14 (42.4)	57 (47.1)	
- No	83	52 (50)	31 (62)		34 (45.9)	49 (61.2)		19 (57.6)	64 (52.9)	
Physical abuse				0.541			0.159			0.522
- Yes	19	14 (13.5)	5 (10)		12 (16.2)	7 (8.8)		3 (9.1)	16 (13.2)	
- No	135	90 (86.5)	45 (90)		62 (83.8)	73 (91.2)		30 (90.9)	105 (86.8)	

*Emotional exhaustion (EE) ≥ 10, depersonalization (D) ≥ 10, low personal accomplishment (PA) <10, and high personal accomplishment ≥ 10*.

* *Significant at p < 0.05*.

For burnout syndrome, working hours per week, residency status, and transport issues were all significantly associated with a higher rate of emotional exhaustion. Meanwhile, none of the variables were significantly associated with depersonalization, indicating a similar distribution between the categorical groups. However, lower personal accomplishment was statistically associated with respondents' years of experience: those with <3 years had higher self-accomplishment, as compared with those with 3–5 years of experience, who perceived themselves as achieving less self-accomplishment. Table 4 demonstrates these differences.

## Limitations

This study has several limitations. First, the sample size allows the study to focus only on one war-torn country, which may have several additional and unique factors that may contribute to high levels of mental distress. These aspects may, in turn, aggravate the effects of the COVID-19 pandemic. Furthermore, interviewer bias may be present, as some respondents may opt to hide or alter their responses out of a fear of stigmatization, despite the anonymous nature of the survey. Additionally, due to the cross-sectional study design, a lower causation and linkage ability may be apparent. In light of these limitations, further studies are required to account for the potential impact of such factors. Moreover, we used self-report evaluation questionnaires that could provide for the level of symptoms; however, they are not used for psychiatric diagnoses and evaluations, which require specific instruments and proper diagnostic methods. To date, no previous studies have focused on emergency physicians residing in countries with civil wars and their mental status during the COVID-19 pandemic.

## Discussion

This study demonstrated higher than expected levels of anxiety, depression, and burnout among 154 emergency doctors from Libya who worked during the COVID-19 pandemic and civil war crisis. Of the respondents, 65.6% reached the cutoff score for anxiety, whereas more than 73% reached the cutoff score for depression. Regarding burnout, about two-thirds (67%) of subjects had emotional exhaustion, while about half (48%) experienced depersonalization, and about 21.4% perceived themselves as having a low level of personal/self-accomplishment.

A higher prevalence and scores of anxiety, depression, and burnout were generally not statistically associated with demographic features. The wider distribution of depression, anxiety, and burnout, despite such differences, can carry dangerous risks related to mental status deterioration, substance abuse, low self-esteem, lower patient care, job dropout or attrition from training, lower sleep quality, a decrease in work satisfaction, and suicidal risks (34–38). Abuse, either verbal or physical, was not significantly different between the groups, as they were exposed to similar risks. The number of working hours per week, however, was associated with emotional exhaustion (as an element of burnout) and anxiety (*p* < 0.05).

This study reveals a very demanding image of the mental health status of emergency physicians working as frontline staff during the COVID-19 pandemic, as foregrounded by civil war (39). It uncovers the prevalence of major mental health disorders, such as anxiety, depression, and burnout, among frontline staff. The female gender was strongly associated with a higher risk of major mental disorders, including general anxiety disorder or mood disorders and post-traumatic stress disorders, mainly in contexts characterized by civil war conflict. However, we found that women with higher degrees of burnout and depressive and anxiety symptoms were not statistically significant. Nevertheless, higher rates of mental disorders were usually associated with women, and there is a high demand for further studies on both health-care workers and the general population in Libya, with an emphasis on other indicators such as post-traumatic stress disorders and severe depressive and anxiety disorders (40, 41).

According to a previous systemic review and meta-analysis of more than 54 studies, the prevalence of depression (between 20.9 and 43.2%) was noted among the studies, based on the characteristics and tools used in depression screening (42). However, our study suggests a 73.4% prevalence of depression, which is higher than in previous studies. Various causes may explain this, such as the public health situation in Libya, which is lacking in social and psychiatric support, and the permeation of civil war and financial crisis in the country, which places a greater burden on physicians who must care for their families while risking death or injury in the war.

In terms of the current COVID-19 situation, the following are some potential reasons for the increase in observed depression: the risk of contamination of health-care workers, the risk of transmitting the infection to family members, a shortage of personal protective equipment (reported as a possible cause of distress), and the lack of adequate training and treatment for COVID-19 management. These are all major stressors that can put physicians at a higher risk for mental distress and produce higher rates of depression, anxiety, and, possibly, burnout syndrome (43–46). Moreover, health-care workers are experiencing additional stressors specific to their jobs, such as work-related stress (e.g., task-specific stressors), working hours, family pressures, the need for specialty training, and a large number of shifts (47).

A study conducted in China by Lai et al. (13) found a 50.4% prevalence of depressive symptoms and a 44.6% prevalence of anxiety using the Patient Health Questionnaire-9 (PHQ-9) tool during the early weeks of the COVID-19 pandemic. Another study conducted by (48) of health-care workers in Spain found a 55.89% prevalence for depressive symptoms and a 67.55% prevalence for anxiety symptoms using the 21-item version of the Depression Anxiety Stress Scales. Their results were similar to our results. Another large study among 5,062 health workers in China found 13.5% depressive symptoms and 24.1% anxiety symptoms using both PHQ-9 and Generalized Anxiety Disorder-7 (GAD-7); the study included 1,004 physicians, 3,417 nurses, and 641 technicians from different specialties (49). However, most of the recently published studies in the literature have focused on general health-care workers, without a specific focus on emergency physicians, who are regarded as frontline physicians during the COVID-19 pandemic.

Our study displayed higher rates of anxiety, depression, and burnout prevalence compared with previously published literature. A study of emergency physicians in Libya before the COVID-19 pandemic found that 45.4% had anxiety symptoms, and the same percentage had depressive symptoms (50). However, we found a higher prevalence in our current study, with anxiety symptoms in 65.6% of patients and depressive symptoms in 73.4%, which may be attributed to the COVID-19 pandemic and an increase in the rate of civil war conflict in early 2020. Another study conducted among physicians of many specialties in Libya found that 56.3% of the participants had depressive symptoms and 46.7% had anxiety symptoms (50). These results show that emergency physicians are at a higher risk of depressive and anxiety symptoms than other medical specialties during the COVID-19 pandemic.

The high prevalence of depression, anxiety, and burnout is a major concern for physicians' well-being and career progress. Several studies have suggested that depression and burnout can carry further risk, progressing chronically throughout a physician's life (51–53). It should also be emphasized that psychological distress and mental fatigue can affect a person's work performance, increasing the risk of medical error, which not only affects physicians but also has adverse effects on the quality of care for patients. This presents a potentially catastrophic effect on the health-care system, increasing the risk for patients as physicians attempt to decrease their working hours or change careers to find less stressful employment (54–56). Burnout affects physicians' and patients' personal lives and has many negative consequences for healthcare-related issues, such as physicians becoming less interested in their work and experiencing mental fatigue, which increases the probability of diagnostic errors, leads to unsafe patient environments, and puts physicians at higher risk of psychological trauma (56).

Several interventions have been suggested as means to decrease the distress experienced by physicians at work, such as the following: use of technology as a reminder to initiate meditation to reduce burnout (57); use of the positive and negative reinforcement strategy outlined by Ratliff et al. which describes the effect of praise on reducing a part of burnout (58); and art therapy, as described by King et al. (59), whereby physicians paint or decorate artistic images, potentially decreasing stress and burnout by focusing on creative outlets. Another interesting approach that could decrease stress is the use of exergame-based rehabilitative interventions that have been proven effective in improving health-related quality of life in chronic diseases (60). Organizational well-being has been proposed as a new method of promoting the psychosomatic health of workers, whereby mental health services are provided in the workplace to health-care workers, based on health system management plans (61).

## Conclusion

Our study indicates that the prevalence of depression, anxiety, and burnout is higher than that reported in previous studies. Physicians' psychological status is crucial and plays a major and influential role in the well-being of health-care workers. An emergency physician's psychological status can affect their work satisfaction; therefore, implementing strategies aimed at decreasing stressful events is crucial in alleviating the distress experienced by physicians on the frontline of the COVID-19 pandemic. These issues will continue to affect those who are on the frontline; therefore, it is essential to work on mental support and services to alleviate their manifestations in the current situation, which is worsening in the country as a result of the ongoing civil war.

## Data Availability Statement

The raw data supporting the conclusions of this article will be made available by the authors, without undue reservation.

## Ethics Statement

The studies involving human participants were reviewed and approved by the Biotechnology Research Center in Libya. The patients/participants provided their written informed consent to participate in this study.

## Author Contributions

ME, AM, and ME analyzed and interpreted the patient data. ME supervised the project. ME wrote the first draft of the manuscript. All authors contributed to the study design, data collection, read, and approved the final manuscript.

## Conflict of Interest

The authors declare that the research was conducted in the absence of any commercial or financial relationships that could be construed as a potential conflict of interest.

## References

[1] Zhou P Yang XL Wang XG Hu B Zhang L Zhang W. A pneumonia outbreak associated with a new coronavirus of probable bat origin. Nature. (2020) 579:270–3. 10.1038/s41586-020-2012-7 32015507 PMC7095418

[2] WHO WHO Director-General's Opening Remarks at the Mission Briefing on COVID-19-16 April 2020. (2020). Available online at: https://www.who.int/dg/speeches/detail/who-director-general-s-opening-remarks-at-the-mission-briefing-on-covid-19 (accessed April 16, 2020).

[3] Dong E Du H Gardner L. An interactive web-based dashboard to track COVID-19 in real time. Lancet Infect Dis. (2020) 20:533–4. 10.1016/S1473-3099(20)30120-1 32087114 PMC7159018

[4] Wong ML Anderson J Knorr T Joseph JW Sanchez LD. Grit, anxiety, and stress in emergency physicians. Am J Emerg Med. (2018) 36:1036–9. 10.1016/j.ajem.2018.02.021 29502975

[5] Shanafelt TD Balch CM Bechamps GJ Russell T Dyrbye L Satele D. Burnout and career satisfaction among American surgeons. Ann Surg. (2009) 250:463–71. 10.1097/SLA.0b013e3181ac4dfd 19730177

[6] Dyrbye LN Massie FS Jr Eacker A Harper W Power D Durning SJ. Relationship between burnout and professional conduct and attitudes among US medical students. JAMA. (2010) 304:1173–80. 10.1001/jama.2010.1318 20841530

[7] Shanafelt TD Balch CM Bechamps G Russell T Dyrbye L Satele D. Burnout and medical errors among American surgeons. Ann Surg. (2010) 251:995–1000. 10.1097/SLA.0b013e3181bfdab3 19934755

[8] Balch CM Shanafelt TD Sloan JA Satele DV Freischlag JA. Distress and career satisfaction among 14 surgical specialties, comparing academic and private practice settings. Ann Surg. (2011) 254:558–68. 10.1097/SLA.0b013e318230097e 21946217

[9] Shanafelt TD Balch CM Dyrbye L Bechamps G Russell T Satele D. Special report: suicidal ideation among American surgeons. Arch Surg. (2011) 146:54–62. 10.1001/archsurg.2010.292 21242446

[10] Oreskovich MR Kaups KL Balch CM Hanks JB Satele D Sloan J Prevalence of alcohol use disorders among American surgeons. Arch Surg. (2012) 147:168–74. 10.1001/archsurg.2011.1481 22351913

[11] Dyrbye LN West CP Hunderfund AL Sinsky CA Trockel M Tutty M. Relationship between burnout, professional behaviors, and cost-conscious attitudes among US physicians. J Gen Intern Med. (2019) 35:1465–76. 10.1007/s11606-019-05376-x 31734790 PMC7210345

[12] Dong L Bouey J. Public mental health crisis during COVID-19 pandemic, China. Emerg Infect Dis. (2020) 26:1616–8. 10.3201/eid2607.202407 32202993 PMC7323564

[13] Lai J Ma S Wang Y Cai Z Hu J Wei N. Factors associated with mental health outcomes among health care workers exposed to coronavirus disease 2019. JAMA Netw Open. (2020) 3:e203976. 10.1001/jamanetworkopen.2020.3976 32202646 PMC7090843

[14] Li Z Ge J Yang M Feng J Qiao M Jiang R. Vicarious traumatization in the general public, members, and non-members of medical teams aiding in COVID-19 control. Brain Behav Immun. (2020) 88:916–9. 10.1101/2020.02.29.20029322 32169498 PMC7102670

[15] Rajkumar RP. COVID-19 and mental health: a review of the existing literature. Asian J Psychiatr. (2020) 52:102066. 10.1016/j.ajp.2020.102066 32302935 PMC7151415

[16] Tan BYQ Chew NWS Lee GKH Jing M Goh Y Yeo LLL. Psychological Impact of the COVID-19 pandemic on health care workers in Singapore. Ann Intern Med. (2020) 173:317–320. 10.7326/M20-1083 32251513 PMC7143149

[17] Wang C Pan R Wan X Tan Y Xu L Ho CS. Immediate psychological responses and associated factors during the initial stage of the 2019 coronavirus disease (COVID-19) epidemic among the general population in China. Int J Environ Res Public Health. (2020) 17:1729. 10.3390/ijerph17051729 32155789 PMC7084952

[18] Xiao H Zhang Y Kong D Li S Yang N. The effects of social support on sleep quality of medical staff treating patients with coronavirus disease 2019 (COVID-19) in January and February 2020 in China. Med. Sci. Monit. (2020) 26:e923549. 10.12659/MSM.923921 32132521 PMC7075079

[19] Michaud K Matheson K Kelly O Anisman H. Impact of stressors in a natural context on release of cortisol in healthy adult humans: a meta-analysis. Stress. (2008) 11:177–97. 10.1080/10253890701727874 18465466

[20] Leblanc VR. The effects of acute stress on performance: implications for health professions education. Acad Med. (2009) 84:S25–33. 10.1097/ACM.0b013e3181b37b8f 19907380

[21] Pizarro J Silver RC Prause J. Physical and mental health costs of traumatic war experiences among civil war veterans. Arch Genrl Psychiatr. (2006) 63:193–200. 10.1001/archpsyc.63.2.193 16461863 PMC1586122

[22] Elhadi M Msherghi A Mental health of surgeons during the COVID-19 pandemic: an urgent need for intervention. Surgery. (2020). 10.1016/j.surg.2020.08.035. [Epub ahead of print]. PMC7489990 33008611

[23] Elhadi M Msherghi A. COVID-19 and civil war in Libya: the current situation. Pathog Glob Health. (2020) 114:230–1. 10.1080/15575330.2020.1769292 32432976 PMC7480619

[24] Snaith RP The hospital anxiety and depression scale. Health Qual Life Outcomes. (2003) 1:29 10.1186/1477-7525-1-29 12914662 PMC183845

[25] Bjelland I Dahl AA Haug TT Neckelmann D. The validity of the hospital anxiety and depression scale an updated literature review *J Psychosom Res*. (2002) 52:69–77. 10.1016/S0022-3999(01)00296-3 11832252

[26] Zigmond AS Snaith RP The hospital anxiety and depression scale. Acta Psychiatr Scand. (1983) 67:361–70. 10.1111/j.1600-0447.1983.tb09716.x 6880820

[27] Maslach C Jackson SE Leiter MP Schaufeli WB Schwab RL. Maslach Burnout Inventory. Palo Alto, CA: Consulting psychologists press (1986).

[28] Mcmanus IC Keeling A Paice E. Stress, burnout and doctors' attitudes to work are determined by personality and learning style: a twelve year longitudinal study of UK medical graduates. BMC Med. (2004) 2:29. 10.1186/1741-7015-2-29 15317650 PMC516448

[29] Maslach C Leiter MP. Early predictors of job burnout and engagement. J Appl Psychol. (2008) 93:498–512. 10.1037/0021-9010.93.3.498 18457483

[30] Mcclafferty H Brown OW. Physician health and wellness. Pediatrics. (2014) 134:830–5. 10.1542/peds.2014-2278 25266440

[31] Waddimba AC Scribani M Nieves MA Krupa N May JJ Jenkins P. Validation of single-item screening measures for provider burnout in a rural health care network. Eval Health Prof . (2016) 39:215–25. 10.1177/0163278715573866 25716107

[32] Riley MR Mohr DC Waddimba AC. The reliability and validity of three-item screening measures for burnout: evidence from group-employed health care practitioners in upstate New York. Stress Health. (2018) 34:187–93. 10.1002/smi.2762 28524379

[33] Von Elm E Altman DG Egger M Pocock SJ Gotzsche PC Vandenbroucke JP. The strengthening the reporting of observational studies in epidemiology. (STROBE) statement: guidelines for reporting observational studies. J Clin Epidemiol. (2008) 61:344–9. 10.1016/j.jclinepi.2007.11.008 18313558

[34] Khoushhal Z Hussain MA Greco E Mamdani M Verma S Rotstein O. Prevalence and causes of attrition among surgical residents: a systematic review and meta-analysis. JAMA Surg. (2017) 152:265–72. 10.1001/jamasurg.2016.4086 27973673

[35] Kuhn CM Flanagan EM. Self-care as a professional imperative: physician burnout, depression, and suicide. Can J Anaesth. (2017) 64:158–68. 10.1007/s12630-016-0781-0 27910035

[36] Kalmoe MC Chapman MB Gold JA Giedinghagen AM. Physician suicide: a call to action. Missouri Med. (2019) 116:211–6. 31527944 PMC6690303

[37] Pereira-Lima K Mata DA Loureiro SR Crippa JA Bolsoni LM Sen S. Association between physician depressive symptoms and medical errors: a systematic review and meta-analysis. JAMA Netw Open. (2019) 2:e1916097. 10.1001/jamanetworkopen.2019.16097 31774520 PMC6902829

[38] Stehman CR Testo Z Gershaw RS Kellogg AR Burnout, drop out, suicide: physician loss in emergency medicine, part I. West J Emerg Med. (2019) 20:485–94. 10.5811/westjem.2019.4.40970 31123550 PMC6526882

[39] Wong AH Pacella-Labarbara ML Ray JM Ranney ML Chang BP. Healing the healer: protecting emergency health care workers' mental health during COVID-19. Ann Emerg Med. (2020) 76:379–84. 10.1016/j.annemergmed.2020.04.041 32534830 PMC7196406

[40] Carta MG Moro D Wallet Oumar F Moro MF Pintus M Pintus E. A follow-up on psychiatric symptoms and post-traumatic stress disorders in tuareg refugees in burkina faso. Front Psychiatr. (2018) 9:127. 10.3389/fpsyt.2018.00127 29740352 PMC5928199

[41] La Cascia C Cossu G Lindert J Holzinger A Zreik T Ventriglio A. Migrant women-experiences from the mediterranean region. Clin Pract Epidemiol Mental Health. (2020) 16:101–8. 10.2174/1745017902016010101 33029187 PMC7536719

[42] Mata DA Ramos MA Bansal N Khan R Guille C Di Angelantonio E. Prevalence of depression and depressive symptoms among resident physicians: a systematic review and meta-analysis. JAMA. (2015) 314:2373–83. 10.1001/jama.2015.15845 26647259 PMC4866499

[43] Joob B Wiwanitkit V. Medical personnel, COVID-19 and emotional impact. Psychiatry Res. (2020) 288:112952. 10.1016/j.psychres.2020.112952 32335465 PMC7152901

[44] Montemurro N. The emotional impact of COVID-19: from medical staff to common people. Brain Behav Immun. (2020) 87:23–4. 10.1016/j.bbi.2020.03.032 32240766 PMC7138159

[45] Rana W Mukhtar S Mukhtar S. Mental health of medical workers in Pakistan during the pandemic COVID-19 outbreak. Asian J Psychiatr. (2020) 51:102080. 10.1016/j.ajp.2020.102080 32283512 PMC7139243

[46] Zhai Y Du X. Addressing collegiate mental health amid COVID-19 pandemic. Psychiatr Res. (2020) 288:113003. 10.1016/j.psychres.2020.113003 32315885 PMC7162776

[47] Joules N Williams D Thompson A Depression in resident physicians: a systematic review. Open J Depression. (2014) 3:89–100. 10.4236/ojd.2014.33013

[48] Odriozola-González P Planchuelo-Gómez Á Irurtia M Luis-García R. Psychological symptoms of the outbreak of the COVID-19 crisis and confinement in the population of Spain. J Health Psychol. (2020). 10.31234/osf.io/mq4fg. [Epub ahead of print]. 33124471

[49] Zhu Z Xu S Wang H Liu Z Wu J Li G. COVID-19 in Wuhan: sociodemographic characteristics and hospital support measures associated with the immediate psychological impact on healthcare workers. EClinicalMedicine. (2020) 24:100443. 10.1016/j.eclinm.2020.100443 32766545 PMC7311903

[50] Elhadi M Khaled A Malek AB El-Azhari AE-A Gwea AZ Zaid A. Prevalence of anxiety and depressive symptoms among emergency physicians in Libya after civil war: a cross-sectional study. BMJ Open. (2020) 10:e039382. 10.1136/bmjopen-2020-039382 32859667 PMC7454180

[51] Girard DE Hickam DH Gordon GH Robison RO. A prospective study of internal medicine residents' emotions and attitudes throughout their training. Acad Med. (1991) 66:111–4. 10.1097/00001888-199102000-00014 1993094

[52] Galletta M Portoghese I Ciuffi M Sancassiani F Aloja ED Campagna M. Working and environmental factors on job burnout: a cross-sectional study among nurses. Clin Pract Epidemiol Ment Health. (2016) 12:132–41. 10.2174/1745017901612010132 27990173 PMC5120375

[53] Carta MG Preti A Portoghese I Pisanu E Moro D Pintus M. Risk for depression, burnout and low quality of life among personnel of a university hospital in Italy is a consequence of the impact one economic crisis in the welfare system? Clin Pract Epidemiol Ment Health. (2017) 13:156–67. 10.2174/1745017901713010156 29238392 PMC5712646

[54] West CP Huschka MM Novotny PJ Sloan JA Kolars JC Habermann TM. Association of perceived medical errors with resident distress and empathy: a prospective longitudinal study. JAMA. (2006) 296:1071–8. 10.1001/jama.296.9.1071 16954486

[55] West CP Tan AD Habermann TM Sloan JA Shanafelt TD. Association of resident fatigue and distress with perceived medical errors. JAMA. (2009) 302:1294–300. 10.1001/jama.2009.1389 19773564

[56] Patel UK Zhang MH Patel K Malik P Shah M Rasul BM. Recommended strategies for physician burnout, a well-recognized escalating global crisis among neurologists. J Clin Neurol. (2020) 16:191–201. 10.3988/jcn.2020.16.2.191 32319235 PMC7174113

[57] Yeo CJJ Barbieri A Roman G Wiesman J Powell S Using smartphone mindfulness apps to increase trainee resilience and reduce burnout. (P2. 9-005). Neurology. (2019) 92(Suppl. 15).

[58] Ratliff J Weissfeld T Skidmore C Southerland A O'donovan C Carrera J Autonomy and praise from co-residents may protect against burnout in neurology residents (P3. 008). Neurology. (2018) 90(Suppl. 15).

[59] King J Walls B Zauber S Hildebrandt A Singhal S Pascuzzi R Art therapy in the management of neurology wellness and burnout-a pilot study (P3. 9-079). Neurology. (2019) 92(Suppl. 15).

[60] Cugusi L Prosperini L Mura G. Exergaming for Quality of Life in persons living with chronic diseases: a systematic review and meta-analysis. PM&R. (2020). 10.1002/pmrj.12444. [Epub ahead of print]. 32592238

[61] Sancassiani F Campagna M Tuligi F Machado S Cantone E Carta MG. Organizational wellbeing among workers in mental health services: a pilot study. Clin Pract Epidemiol Ment Health. (2015) 11:4–11. 10.2174/1745017901511010004 25767557 PMC4353129

